# The Threshold Effect: Lipopolysaccharide-Induced Inflammatory Responses in Primary Macrophages Are Differentially Regulated in an iRhom2-Dependent Manner

**DOI:** 10.3389/fcimb.2020.620392

**Published:** 2021-01-29

**Authors:** Joseph Skurski, Garima Dixit, Carl P. Blobel, Priya D. Issuree, Thorsten Maretzky

**Affiliations:** ^1^ Inflammation Program, Roy J. and Lucille A. Carver College of Medicine, Department of Internal Medicine, University of Iowa, Iowa City, IA, United States; ^2^ Immunology Graduate Program, Roy J. and Lucille A. Carver College of Medicine, Department of Internal Medicine, University of Iowa, Iowa City, IA, United States; ^3^ Departments of Medicine and of Physiology, Biophysics and Systems Biology, Weill Cornell Medicine, New York, NY, United States; ^4^ Arthritis and Tissue Degeneration Program, Hospital for Special Surgery, New York, NY, United States; ^5^ Molecular Medicine Graduate Program, Roy J. and Lucille A. Carver College of Medicine, Department of Internal Medicine, University of Iowa, Iowa City, IA, United States; ^6^ Holden Comprehensive Cancer Center, Roy J. and Lucille A. Carver College of Medicine, Department of Internal Medicine, University of Iowa, Iowa City, IA, United States

**Keywords:** a disintegrin and metalloproteinase 17, ADAM17, inactive rhomboid 2, iRhom2, lipopolysaccharide, LPS, tumor necrosis factor, TNF

## Abstract

A well-controlled innate immune response is characterized by a rapid yet self-limiting inflammatory response. Although much is known about the range of inflammatory stimuli capable of triggering an innate immune response, the mechanisms which govern the degree of inflammation induced by inflammatory insults and the mechanisms in place to reset or maintain homeostasis are poorly understood. Tumor necrosis factor (TNF) is a potent early response pro-inflammatory cytokine produced by immune cells following a broad range of insults spanning autoimmunity and metabolic diseases to pathogenic infections. Previous studies have shown that a disintegrin and metalloproteinase (ADAM) 17 controls the release of soluble TNF and epidermal growth factor receptor signaling. Utilizing a genetic model of ADAM17 deficiency through the deletion of its regulator, the inactive rhomboid 2 (iRhom2), we show that loss of ADAM17 activity in innate immune cells leads to decreased expression of various cytokines in response to low levels of pathogen-associated molecular pattern (PAMP) stimulation but not at high-dose stimulation. In addition, *TNF receptor (TNFR*) *1/2*-deficient bone marrow-derived macrophages yielded significantly reduced TNF expression following low levels of PAMP stimulation, suggesting that signaling through the TNFRs in immune cells drives a feed-forward regulatory mechanism wherein low levels of TNF allow sustained enhancement of TNF expression in an iRhom2/ADAM17-dependent manner. Thus, we demonstrate that inflammatory expression of *TNF* and *IL1β* is differentially regulated following high or low doses of PAMP stimulation, invoking the activation of a previously unknown regulatory mechanism of inflammation.

## Introduction

Aberrant tumor necrosis factor (TNF) signaling is a key pathogenic player in multiple chronic inflammatory diseases, including rheumatoid arthritis, lupus nephritis, and bacterial infection (sepsis) (Spooner et al., 1992; Mageed and Isenberg, 2002; Issuree et al., 2013; Lichtenthaler, 2013; Geesala et al., 2019). TNF signaling is regulated by the activity of a disintegrin and metalloproteinase (ADAM) 17, a metalloproteinase expressed upon the cell surface, which controls the release of soluble TNF from its membrane-bound form (Black et al., 1997; Moss et al., 1997; Peschon et al., 1998). The inactive rhomboid protein 2 (iRhom2) was recently identified as a crucial regulator of ADAM17 activation in immune cells, thereby regulating the release of soluble TNF (Adrain et al., 2012; McIlwain et al., 2012; Siggs et al., 2012). iRhom2-dependent TNF release has been shown to modulate the inflammatory response in various mouse models of inflammatory disease (McIlwain et al., 2012; Issuree et al., 2013; Haxaire et al., 2018; Qing et al., 2018; Geesala et al., 2019; Skurski et al., 2020). *iRhom2*-deficient (^-/-^) mice show reduced serum TNF in response to LPS, and are able to survive a lethal LPS challenge (McIlwain et al., 2012). Additionally, *iRhom2* ^-/-^ mice fail to control the replication of *Listeria monocytogenes*, suggesting that iRhom2 is required for TNF-mediated antibacterial activity (McIlwain et al., 2012). iRhom2 has also been shown to modulate ADAM17-mediated TNF and EGFR ligand shedding and crosstalk (McIlwain et al., 2012; Christova et al., 2013; Maretzky et al., 2013; Li et al., 2015). However, the potential role for iRhom2 to modulate the expression of other cytokines in immunologically relevant contexts has not been explored.

The present study aimed to investigate the potential role of iRhom2 in the regulation of various pro-inflammatory cytokines in murine macrophages upon pattern recognition receptor stimulation. Given that iRhom2 controls the ADAM17-mediated release of several soluble factors from innate immune cells, including TNF and interleukin 6 receptor (IL6R) that modulate the progression of inflammation *in vivo* (Chalaris et al., 2010), we asked whether and which macrophage cell-driven inflammatory responses are modulated in an iRhom2-dependent manner following pathogen-associated molecular pattern (PAMP) recognition using an *ex vivo* system. Strikingly, we found that iRhom2 acts as an amplifier of the transcriptional inflammatory response following low doses of PAMP challenge; however, high doses of PAMP stimulation triggers a strong inflammatory response independent of iRhom2 in macrophages. Additionally, we demonstrate that low-dose induced expression of early response pro-inflammatory cytokine genes, including TNF itself, are significantly blunted in *TNF receptor* (*TNFR*) *1/2* ^-/-^ (*TNFRSF1A/TNFRSF1B* ^-/-^) and *TNF* ^-/-^ bone marrow-derived macrophages (BMDMs), highlighting a critical role for soluble TNF as a coordinator of the inflammatory response under low PAMP challenge. Taken together, our studies indicate the presence of a novel, previously uncharacterized inflammatory pathway wherein iRhom2 mediates the innate immune response through the expression of inflammatory cytokines in a dose-dependent manner.

## Materials and Methods

### Mice

Age-matched C57BL/6 wild type (WT), *iRhom2* ^-/-^, *TNF* ^-/-^, *TNFRSF1A* ^-/-^, and *TNFRSF1A/TNFRSF1B* ^-/-^ mice were used for all experiments. *iRhom2* ^-/-^ mice obtained from Dr. Tak Mak’s lab at the Campbell Family Institute for Breast Cancer Research were described previously (McIlwain et al., 2012). *TNF* ^-/-^, *TNFRSF1A* ^-/-^, and *TNFRSF1A/B* ^-/-^mice were obtained from the Jackson Laboratory and maintained in our facility. All mice were bred in specific pathogen-free conditions at the University of Iowa Animal Resource Center. All experiments were performed in accordance with the University of Iowa Institutional Animal Care and Use Committee (IACUC) Guidelines.

### Reagents and Inhibitors

All reagents were endotoxin-free (*Limulus amoebocyte* lysate-Pyrochrome kit, Associates of Cape Cod, Inc., East Falmouth, MA, USA). CL264 was from EMD Millipore (Burlington, MA, USA), and *Escherichia coli* lipopolysaccharide (LPS) was from Chemicon International (Temecula, CA, USA). The metalloproteinase inhibitor DPC333 was kindly provided by the Hospital for Special Surgery, New York, NY (Qian et al., 2007; Le Gall et al., 2010). A function blocking anti-TNF monoclonal antibody (Clone TN3-19.12) was purchased from BioXCell (Lebanon, NH, USA) and recombinant TNF was purchased from Pepro Tech (Rocky Hill, NJ, USA). All other reagents were purchased from Sigma Aldrich (St. Louis, MO, USA), unless otherwise stated.

### Cell Culture

Bone marrow-derived macrophage cells (BMDMs) from femoral and tibial bone marrow were propagated in the presence of macrophage colony-stimulating factor (M-CSF). This macrophage growth factor was secreted by L929 cells and used in the form of L929 cell conditioned medium. BMDMs were cultured in DMEM containing 10% heat-inactivated fetal bovine serum (FBS), 30% L929 conditioned medium, 1% non-essential amino acids, and 1% penicillin-streptomycin. Fresh medium was added on days 3, 5, and 7 prior to all experiments.

### Transcriptional Response Assays

Isolated murine BMDMs were seeded into 6-well plates at a density of 3.0×10^6^ cells per well in 1 ml of L929 conditioned medium overnight. BMDMs were then starved of L929 in 900 μl of culture medium containing RMPI 1640 with 10% FBS and penicillin/streptomycin for 4 h. BMDMs were then treated with 10 μl s of LPS diluted in culture medium at a final concentration of 0.002, 0.02, 0.2, 2, 200, or 2,000 ng/ml. Treatment was over the course of up to 4 h. An equivalent volume of culture medium was included as a negative control.

### RT-qPCR

Isolated BMDMs were subjected to mechanical homogenization in RLT buffer, and mRNA was isolated using a RNeasy Mini Kit according to the manufacturer’s protocol (Qiagen, Hilden, Germany). mRNA was then reverse transcribed into cDNA using RevertAidM-MuLV (Thermo Fisher, MA, USA), reverse transcriptase, which was utilized for RT-qPCR using a SYBR green master mix on an Eppendorf (Hamburg, Germany) RealPlex thermocycler. KiCqStart primer sets for *TNF*, *IL1β*, *IL13*, *CXCL2*, *IL10*, *IL4, ACTB*, and *TBP* were purchased from MilliporeSigma (Burlington, MA, USA). *TBP* and *ACTB* were used as reference genes for all RT-qPCR experiments, and gene expression values were calculated using the 2^-ΔΔ^CT method.

### Protein Expression Analysis

Soluble TNF levels were quantified in cell culture medium using a sandwich ELISA (Thermo Fisher, MA, USA). Soluble IL10 levels were quantified in cell culture medium by simplex sandwich ELISA according to the manufacturer’s protocols (Thermo Fischer, MA, USA). Absorbance values from the IL10 analysis were normalized to the highest recorded values (100%) to present changes in relative protein production.

### Flow Cytometry

Monoclonal antibodies were purchased from BioLegend. Clones were anti-CD120a (TNFR1, 1:200), anti-CD120b (TNFR2, 1:200), anti-CD284 (TLR4, 1:200), Rat IgG2a (isotype control 1:200), and Armenian Hamster IgG (isotype control 1:200). Cell viability dye BV Ghost 150 (1:300) was used to exclude dead cells. Flow cytometry was performed using a CtyoFLEX (Beckman Coulter, CA, USA) and data were analyzed with FlowJo v10.7.

### Statistical Analysis

Values are expressed as means ± standard error of the mean (s.e.m.). The standard error values indicate the variation between mean values obtained from three independent samples per group. Statistical analyses were performed using an unpaired, two‐tailed student’s *t* test. Multiple comparisons between experimental groups were analyzed by two‐way ANOVA. Multiple parametric statistical comparisons between experimental groups versus a control group were accomplished by Dunnett’s method. Pairwise multiple comparison procedures were performed *via* the Bonferroni’s multiple comparison’s test. *P* values of ≤0.05 were considered statistically significant.

## Results

### Metalloproteinase Inhibition in Murine BMDMs Blunts Low-Dose LPS-Stimulated Expression of *TNF* and *IL1β*

**Figure 1:**
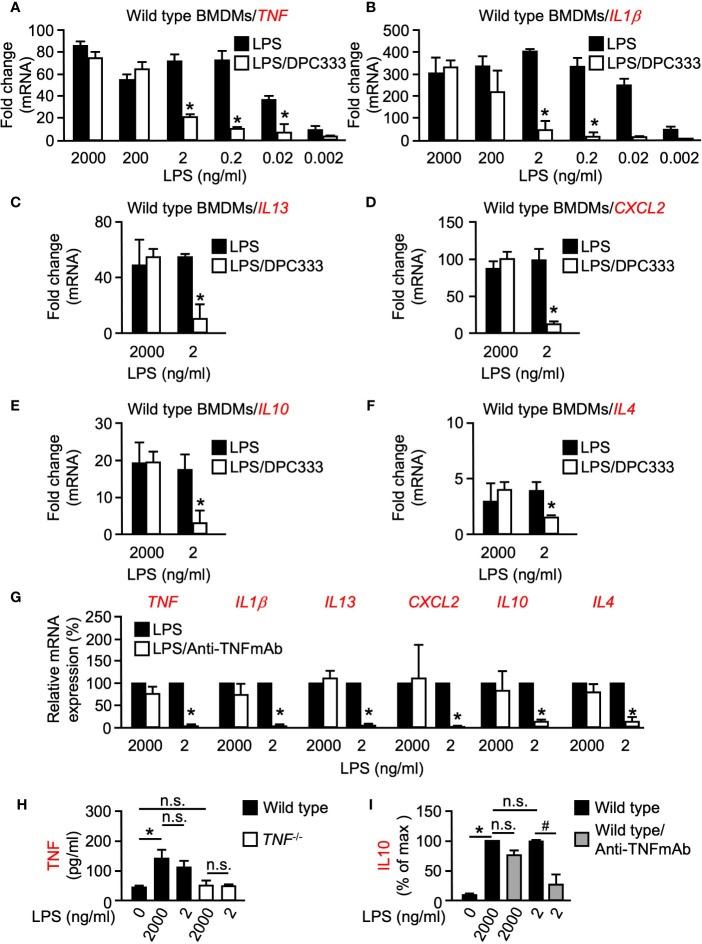
LPS-induced expression of multiple genes is differentially regulated at low and high doses of LPS in BMDMs in a metalloproteinase-dependent manner. Quantification of the mRNA levels of *TNF* **(A)** and *IL1β* **(B)** by RT-qPCR in murine WT BMDMs following stimulation with titrated doses of LPS for 1 h in the presence or absence of 5 µM of the metalloproteinase inhibitor DPC333. Quantification of the mRNA levels of *IL13* **(C)**, *CXCL2* **(D)**, *IL10* **(E)**, and *IL4* **(F)** by RT-qPCR in WT BMDMs following stimulation with 2 ng/ml (low dose) and 2,000 ng/ml (high dose) LPS for 1 h in the presence or absence of 5 µM of the metalloproteinase inhibitor DPC333. An asterisk (*) indicates a significant decrease in gene expression in the DPC333-treated sample compared with the sample without DPC333 **(A–F)**. **(G)** Quantification of mRNA levels of *TNF*, *IL1β*, *IL13*, *CXCL2*, *IL10*, and *IL4* by RT-qPCR in WT BMDMs following 1 h pre-treatment with or without 10 μg/ml Anti-TNFmAb and stimulation with 2 ng/ml (low dose) and 2,000 ng/ml (high dose) LPS for 1 h, in the presence or absence of 10 μg/ml anti-TNFmAb. The * indicates a significant decrease in gene expression in the anti-TNFmAb-treated sample compared with the sample without anti-TNFmAb. **(H)** WT BMDMS were stimulated with low-dose or high-dose LPS for 1 h, with *TNF^-/-^* BMDMS serving as negative control. Soluble TNF in the culture supernatant was measured by ELISA. **(I)** WT BMDMS were treated with or without 10 μg/ml anti-TNFmAb and stimulated with low-dose or high-dose LPS for 1 h. Soluble IL10 in the culture supernatant was quantified by absorbance and normalized as a percentage of the highest recorded values. An * indicates a significant increase in soluble TNF **(H)** or IL10 **(I)** in the LPS-treated sample compared with the unstimulated sample while a ^#^ indicates a significant decrease in soluble IL10 in the anti-TNFmAb-treated sample compared with the sample without anti-TNFmAb. *n* = 3; ± s.e.m; Bonferroni’s multiple comparison’s test, ^#^ and *P ≤ 0.05, not significant (n.s.).

To investigate a potential role of metalloproteinase activity in the transcriptional regulation of pro-inflammatory cytokines, BMDMs were isolated from WT mice and stimulated with increasing concentrations of LPS [a potent inducer of inflammation found in the cell wall of gram-negative bacteria, (Xaus et al., 2000)] for 1 h, in the presence or absence of the hydroxamate-based ADAM17 metalloproteinase inhibitor DPC333 (Qian et al., 2007). Metalloproteinase inhibition had no significant effect on the expression of *TNF* or *IL1β* at 2,000 ng/ml of LPS stimulation (**Figures 1A, B**). Intriguingly, this pattern was not observed at the lower-doses of LPS stimulation whereby metalloproteinase inhibition led to a significant decrease in the expression of both *TNF* and *IL1β* when BMDMs were stimulated with 2 ng/ml LPS or less (**Figures 1A, B**). These findings suggest that metalloproteinase activity is dispensable for the expression of both *TNF* and *IL1β* following high levels of inflammatory stimulus; however, metalloproteinase activity is necessary for the expression of these genes following low levels of inflammatory stimulus.

### LPS-Induced Expression of Multiple Genes Is Differentially Regulated at Low and High Doses of LPS in BMDMs in a Metalloproteinase-Dependent Manner

Following our observation that metalloproteinase activity plays a differential regulatory role between high and low doses of inflammatory stimulus in early response pro-inflammatory genes, we next sought to determine whether metalloproteinase activity controls a similar regulatory arm among other inflammation-related genes. Multiple pro-inflammatory genes including *TNF*, *IL1β*, and *IL13* yielded significant differences in expression between high and low-dose LPS treatment in the presence of DPC333 (**Figures 1A–C**). We next assessed the metalloproteinase dependence of an important chemokine, CXCL2, which regulates macrophage chemotaxis early in the inflammatory response (De Filippo et al., 2013). *CXCL2* expression also showed a dose- and metalloproteinase-dependent regulation, wherein low-dose LPS treatment drove a metalloproteinase-dependent regulatory response that was not observed following high-dose LPS treatment (**Figure 1D**). Furthermore, *IL10* and *IL4* expression remained unaffected by metalloproteinase inhibition following high levels of LPS stimulation, while the expression levels for each of these genes decreased significantly when treated with DPC333 upon stimulation with low-dose LPS (**Figures 1E, F**). Overall, these results suggest that the expression of multiple genes involved in both pro- and anti-inflammatory regulatory responses are differentially regulated in a metalloproteinase-dependent manner, based upon the degree of inflammatory stimulus.

We next sought to determine the role of TNF signaling on the expression of these cytokines following low and high-dose LPS stimulation. We observed significant decreases in the expression of all cytokines tested following low-dose LPS stimulation in the presence of a function blocking TNF monoclonal antibody (Anti-TNFmAb, **Figure 1G**), suggesting that the metalloproteinase-dependent regulation of these genes following low-dose LPS stimulation is likely regulated by TNF signaling. In addition, we observed that both low and high doses of LPS resulted in similar TNF secretion from WT BMDMs, recapitulating our observations regarding *TNF* mRNA expression (**Figure 1H**). To determine whether the observed TNF-dependent differential regulation translates to the protein level, we analyzed secreted IL10 production from WT BMDMs following low and high-dose LPS stimulation in the presence or absence of anti-TNFmAb. We observed significant decreases in IL10 production following low-dose LPS stimulation in the presence of anti-TNFmAb that was not recapitulated following high-dose LPS stimulation (**Figure 1I**). Taken together, these results demonstrate that the metalloproteinase-dependent differential regulatory mechanism is likely regulated by TNF in response to inflammatory stimuli.

### LPS-Induced Expression of *IL1β* and *TNF* Is Regulated by TNF at Low Doses of LPS in Murine BMDMs

**Figure 2:**
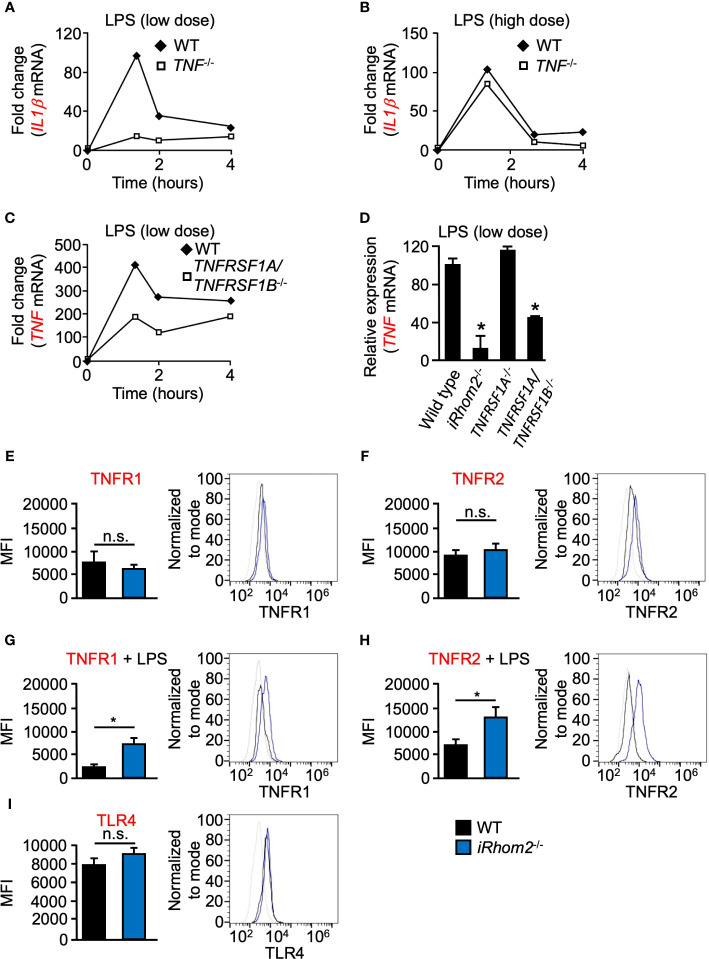
LPS-induced expression of *IL1β* and *TNF* is regulated by TNF/TNFR at low doses of LPS in murine BMDMs. Quantification of the mRNA levels of *IL1β* **(A, B)** and *TNF* **(C, D)** by RT-qPCR in WT **(A–D)**, *TNF* ^-/-^ **(A, B)**, *TNFRSF1A/TNFRSF1B^-/-^* **(C, D)**, or *TNFRSR1A* ^-/-^ **(D)** BMDMS following stimulation with 2 ng/ml (low dose; **A, C, D**) and 2,000 ng/ml (high dose; **B**) LPS for the indicated time points. One representative of three independent experiments with two technical replicates per experiment is shown **(A–C)**. An asterisk (*) in panel D indicates a significant decrease in *TNF* gene expression in the *TNFRSF1A/TNFRSF1B^-/-^* or *TNFRSF1A* ^-/-^ sample compared with the WT sample. *n* = 3; ± s.e.m; Dunnett’s method, *P ≤ 0.05. Membrane-bound TNFR1 **(E, G)**, TNFR2 **(F, H)** and TLR4 **(I)** expression was determined in WT and *iRhom2^-/-^* BMDMS by flow cytometry. Isolated WT and *iRhom2^-/-^* BMDMs were stimulated with 2,000 ng/ml LPS for 1 h, and expression of TNFR1 **(G)** and TNFR2 **(H)** was assayed by flow cytometry. One representative histogram of three independent experiments (2 mice per group) with two technical replicates per experiment is shown **(E–I)**. The * in panels **(G, H)** indicate a significant decrease in cell surface expression of TNFR1 **(G)** or TNFR2 **(H)** in the in the LPS-treated sample compared with the unstimulated sample. *n* = 3; ± s.e.m; Student’s *t*-test, *P ≤ 0.05, not significant (n.s.).

Given the requirement of metalloproteinase activity for TNF expression following low doses of LPS stimulation, we next tested the hypothesis that TNF signaling *via* TNFRs may produce a feed-forward signal which serves to enhance TNF expression in a metalloproteinase-dependent manner. To test the hypothesis that TNF is indeed the ligand responsible for this proposed feed-forward loop, we quantified *IL1β* expression in *TNF^-/-^* BMDMs. We found that *TNF^-/-^* BMDMs had a lower level of *IL1β* expression compared to WT cells, following low-dose LPS stimulation over a 4-h time course (**Figure 2A**). We did not observe significant differences in *IL1β* expression in *TNF^-/-^* BMDMs following high-dose LPS stimulation, suggesting that the degree of stimulus alone invokes a differential regulatory response independent of time (**Figure 2B**). Signaling through TNFRs has been shown to be a key mediator for the induction of the TNF-mediated inflammatory response (Kondo and Sauder, 1997). To further establish that shed TNF can signal in an autocrine/paracrine manner to amplify gene expression, we used *TNFR1/2* (*TNFRSf1A/TNFRSF1B*)^-/-^ mice. *TNFR1/2^-/-^* BMDMs yielded significantly less *TNF* expression than WT BMDMs for up to 4 h, suggesting that TNFR signaling is necessary for *TNF* expression early in the inflammatory response (**Figure 2C**). Interestingly, however, in contrast to *TNFR1/2^-/-^* BMDMs, *TNF* expression in *TNFR1^-/-^* BMDMs was comparable to WT levels suggesting a potential role for TNFR2 in a mediating a TNF-signaling feed-forward process (**Figure 2D**).

iRhoms are upstream regulators of ADAM17, and deficiency of ADAM17 or combined deletion of iRhoms 1 and 2 in mice results in perinatal death (Peschon et al., 1998; Li et al., 2015), while *iRhom2* ^-/-^ mice are healthy and display no spontaneous pathological phenotype (Adrain et al., 2012; McIlwain et al., 2012; Siggs et al., 2012) presumably due to the redundant role of iRhom1 in other tissues (Li et al., 2015). However, since most immune cells selectively express iRhom2, ADAM17 activity is abrogated in *iRhom2* ^-/-^ macrophages (Adrain et al., 2012; McIlwain et al., 2012; Issuree et al., 2013). Therefore, we utilized BMDMs from *iRhom2* ^-/-^ mice to determine whether iRhom2 regulates *TNF* expression through a feed-forward loop. When treated with a low dose of LPS, *iRhom2* ^-/-^ BMDMs displayed a significantly reduced TNF expression compared to WT controls (**Figure 2D**).

We next asked whether TNFR1 or TNFR2 expression on the cell surface differs between WT and *iRhom2* ^-/-^ BMDMs. Flow cytometric analysis revealed no significant differences in TNFR1 or TNFR2 expression between WT and *iRhom2* ^-/-^ BMDMs at baseline; however, stimulation with LPS for 1 h resulted in significant decreases in TNFR1 and TNFR2 expression on the cell surface of WT BMDMs (**Figures 2E–H**). These results demonstrated that TNFR1 and TNFR2 cell surface expression is regulated in an iRhom2-dependent manner following LPS stimulation. We further observed no significant difference in TLR4 expression between WT and *iRhom2* ^-/-^ BMDMs, suggesting that the observed metalloproteinase-dependent regulation of the cytokines shown above is independent of differences in TRL4 expression (**Figure 2I**). Taken together, our data suggest the existence of a feed-forward mechanism that drives *TNF* expression and other cytokines in an iRhom2- and presumably ADAM17-dependent manner in BMDMs when challenged with a low-dose of LPS.

### LPS-Induced Expression of TNF and *IL1β* Is Differentially Regulated at Low and High Doses of LPS in *iRhom2* ^-/-^ BMDMs

**Figure 3:**
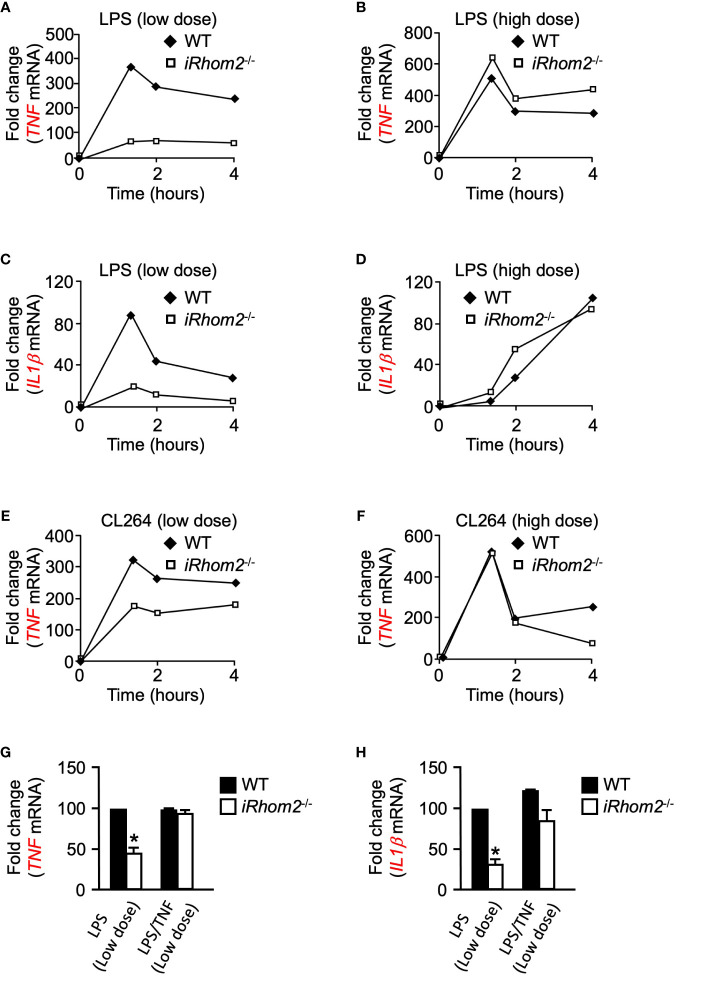
Toll-like receptor ligands-induced expression of *IL1β* and *TNF* depends on iRhom2 at low doses of LPS or CL264 in murine BMDMs. Quantification of the mRNA levels of *TNF* by RT-qPCR in WT and *iRhom2^-/-^* BMDMS following stimulation with 2 ng/ml (low dose; **A**) of LPS, or 2,000 ng/ml (high dose; **B**) LPS following stimulation for the indicated time points. Quantification of mRNA levels of *IL1β* by RT-qPCR in WT and *iRhom2^-/-^* BMDMS following stimulation with 2 ng/ml **(C)** of LPS, or 2,000 ng/ml **(D)** LPS following stimulation for the indicated time points. Quantification of mRNA levels of *TNF* by RT-qPCR in WT and *iRhom2^-/-^* BMDMS following stimulation with 2 ng/ml (low dose; **E**) of CL264, or 2,000 ng/ml (high dose; **F**) CL264 following stimulation for the indicated time points. One representative of three independent experiments with two technical replicates per experiment is shown **(A–F)**. Quantification of the mRNA levels of *TNF* **(G)** and *IL1β* **(H)** by RT-qPCR in WT and *iRhom2^-/-^* BMDMs following stimulation with 2 ng/ml or 2,000 ng/ml LPS for 1 h. One ng/ml of recombinant TNF was added 7 min following activation with LPS. An asterisk (*) in panels G and H indicates a significant decrease in *TNF* gene expression in the *iRhom2^-/-^* sample compared with the WT sample. *n* = 3; ± s.e.m; Bonferroni’s multiple comparison’s test, *P ≤ 0.05.

We next asked whether iRhom2 regulates the expression of other pro-inflammatory genes in a dose- and time-dependent manner following inflammatory stimulation. BMDMs from *iRhom2^-/-^* mice stimulated with low-dose LPS displayed significantly lower expression levels of *TNF* than WT BMDMs for up to 4 h. (**Figure 3A**). Intriguingly, *iRhom2^-/-^* BMDMs stimulated with a high-dose LPS treatment for up to 4--h displayed no significant difference in *TNF* expression in comparison to WT BMDMs (**Figure 3B**). We observed a similar dose-dependent differential regulatory pattern with respect to the expression of *IL1β*. BMDMs from *iRhom2^-/-^* mice stimulated with a low-dose LPS expressed significantly lower levels of *IL1β* than *iRhom2^-/-^* BMDMs stimulated with a high-dose LPS for up to 4 h. (**Figures 3C, D**). Notably, high doses of LPS stimulation led to gradual increases in *IL1β* expression over time, while low levels of LPS stimulation produced a sharp, transient increase in expression (**Figures 3C, D**). Taken together, these results suggest that iRhom2 may play a crucial role in the regulation of the early inflammatory response following low levels of stimulus.

### CL264-Induced Expression of TNF Is Differentially Regulated at Low and High Doses of LPS in *iRhom2^-/-^* BMDMs

We next sought to determine whether the differentially regulated iRhom2 and metalloproteinase-dependent inflammatory gene response could be replicated through stimulation of other toll-like receptor (TLR) ligands. We treated WT and *iRhom2* ^-/-^ BMDMs with the TLR7 agonist CL264 for a 4-h interval and quantified *TNF* expression. Similar to the effects observed following LPS stimulation, *iRhom2* ^-/-^ BMDMs displayed less *TNF* expression compared to WT BMDMs in response to a low dose (2 ng/ml) of CL264 (**Figure 3E**). In contrast, no significant differences in early response *TNF* expression were observed between WT and *iRhom2* ^-/-^ BMDMs at higher doses (2,000 ng/ml) of CL264, further suggesting that low levels of inflammatory stimulus are regulated in an iRhom2/ADAM17-dependent manner (**Figure 3F**).

### *TNF* Expression Is Driven in an iRhom2-Dependent Feed-Forward Mechanism Following Low Doses of Inflammatory Stimulus

We next asked whether TNF can induce its own expression in a feed-forward manner. To address this question, we stimulated WT and *iRhom2* ^-/-^ BMDMs with low doses of LPS in the presence of a low concentration of recombinant TNF. We observed that the significant reduction in *TNF* expression in *iRhom2* ^-/-^ BMDMs could be rescued by the addition of recombinant TNF (**Figure 3G**). We observed the same phenotype in *IL1β* expression (**Figure 3H**). Taken together, these results further corroborate our hypothesis that *TNF* expression is driven in an iRhom2-dependent feed-forward mechanism following low doses of inflammatory stimulus, and that other inflammatory cytokines such as *IL1β* can be regulated in a similar manner.

## Discussion

Subclinical levels of circulating endotoxin are associated with immune cell-mediated, chronic, low-grade inflammation and have been implicated in an array of pathologies, including rheumatoid arthritis and obesity (Issuree et al., 2013; Skurski et al., 2020). Given our recent observation that bacteria/endotoxin exposure causes a low-grade inflammatory state in the intestine of *iRhom2/Il10* ^-/-^ mice arising from a presumably more permeable intestinal wall (Geesala et al., 2019), we sought to determine how iRhom2/metalloproteinase-driven low-grade inflammatory mechanisms may be regulated. At low doses of LPS stimulation, *TNF* and *IL1β* expression was reduced in the presence of the hydroxamate-based ADAM17 metalloproteinase inhibitor DPC333, suggesting the presence of a physiological threshold wherein metalloproteinase activity is required for *TNF* and *IL1β* expression. Intriguingly, at high doses of LPS stimulation, *TNF* and *IL1β* appear to be transcriptionally regulated in a metalloproteinase-independent manner. These results suggest that a metalloproteinase, most likely ADAM17, may serve as a threshold regulator for the transcriptional activity of key inflammatory cytokine genes in response to relatively low levels of inflammatory stimuli (Manicone and McGuire, 2008). Interestingly, although dose strength can regulate the kinetics of gene expression such as *IL1β*, the length of stimulation appears to have no effect upon the induction of this differentially regulated pathway under low-dose conditions, suggesting that the level of stimulus alone determines whether metalloproteinase activity is needed for the transcriptional regulation of *TNF* and *IL1β*. Importantly, we recently showed using the G protein-coupled receptor-dependent stimulus C5a, that dose-dependent stimulation of human peripheral blood mononuclear cells with C5a induced the expression of *TNF*, *IL1β*, and *CXCL2* in a metalloproteinase-dependent manner, highlighting the conservation of this mode of gene regulation in primary immune cells (Skurski et al., 2020).

It is known that macrophage-derived MMP12 cleaves and inactivates soluble CXCL2, which reduces chemotaxis of neutrophils and other macrophages, thereby modulating the innate immune response (Dean et al., 2008). We demonstrate that *CXCL2* is also differentially regulated at a transcriptional level in a metalloproteinase-dependent manner following low-dose LPS treatment, yet the transcriptional expression of *CXCL2* is unaffected by metalloproteinase inhibition following high-dose LPS treatment. These results suggest that different classes of metalloproteinases regulate the early inflammatory response through the production of inflammatory mediators such as *TNF* and *IL1β*, but also by regulating their half-life, such as CXCL2. Low levels of inflammatory stimuli may be regulated by metalloproteinase-mediated trafficking of neutrophils and macrophages to the site of inflammatory challenges. During a highly inflammatory insult, metalloproteinase activity may be bypassed to favor a more expedient mechanism for cytokine production and leukocyte trafficking (Van Lint and Libert, 2007).

Additionally, we demonstrate that anti-inflammatory cytokines including *IL10* are differentially regulated in a similar manner. Th2-associated *IL4* and *IL13* are also subject to metalloproteinase-dependent transcriptional regulation following low-dose LPS challenge, suggesting that a broad range of immunologic insults are differentially regulated through metalloproteinase activity. Recent studies have shown that IL4 signaling mediates the polarization of tissue-resident macrophages to an anti-inflammatory phenotype, contributing to an anti-inflammatory microenvironment that facilitates wound healing (Jenkins et al., 2013). Similarly, macrophage-derived IL10 plays a crucial role in intestinal mucosal wound repair (Quiros et al., 2017). Previous studies have shown that IL10 can be generated by macrophages in response to TLR signaling following LPS stimulation, suggesting that macrophage-derived IL10 may play a key role in resolving acute inflammation to avoid excessive tissue damage (Iyer et al., 2010). In our model, *IL10* expression is differentially regulated *via* metalloproteinase activity, suggesting that highly inflammatory insults may bypass metalloproteinase-dependent regulation, allowing IL10 to reduce localized inflammation to preserve tissue integrity during the innate immune response. It is tempting to speculate that basal levels of inflammatory stimuli require the activation of metalloproteinases as an inflammatory safeguard, which may favor an anti-inflammatory (M2-like) polarization to reduce localized inflammation in tissues (Saqib et al., 2018). However, further studies are required to test the notion that the polarization of macrophages toward the M2-like phenotype is regulated in an iRhom2-dependent manner under low levels of inflammation.

One possible explanation for our observations that inflammatory cytokines are differentially regulated in a metalloproteinase-dependent manner, could be that iRhom2 provides a physiological threshold in macrophages, where low levels of inflammation require metalloproteinase-dependent, and thus most likely ADAM17-dependent TNF shedding to regulate the strength of the innate immune response. Highly inflammatory insults may bypass the iRhom2-dependent cytokine production which results in a feed-forward mechanism to amplify the recruitment of neutrophils and other macrophages to the site of injury. Such a differential regulatory mechanism may be advantageous for the modulation of the immune response to basal levels of inflammatory stimuli. It is possible that additional cytokines beyond TNF, including IL6, may contribute to our observed feed-forward regulation. However, this regulation may operate later in the inflammatory response given the kinetics of expression, although additional studies are needed to determine the involvement of other inflammatory mediators in a comprehensive manner. Following a low-dose LPS treatment, we observed that the addition of recombinant TNF rescues the reduced *TNF* expression in *iRhom2* ^-/-^ BMDMs, suggesting that iRhom2/ADAM17-mediated shedding of soluble TNF early during LPS activation acts in a feed-forward manner to regulate its own transcription. It is possible that the early source of TNF derives from low levels of preexisting transmembrane TNF. Previous reports have shown that the inhibition of soluble TNF signaling in a murine model of inflammatory bowel disease has little therapeutic effect; however, the inhibition of transmembrane TNF results in remission (Perrier et al., 2013). These results implicate transmembrane TNF signaling in the development of inflammatory pathology independent of the soluble form, at least in this model (Perrier et al., 2013). Indeed, *iRhom2* ^-/-^/*IL10* ^-/-^ mice develop a more severe spontaneous colitis than *IL10* ^-/-^ mice alone (Geesala et al., 2019). Given these observations, it is possible that highly potent inflammatory insults could stimulate an iRhom2/ADAM17-independent induction of cytokine expression through the remaining transmembrane TNF. However, our *in vitro* studies suggest that transmembrane TNF signaling does not play a crucial role for the induction of cytokine mRNA expression, such as *IL1β*, following low-dose stimulation.

The commensal microbiota in the human gut is a rich source of bacterial antigen (Steimle et al., 2016). Pro-inflammatory stimuli such as LPS presented by bacteria in the gut may induce low levels of TLR4 engagement (Kim et al., 2012). This would lead to only basal levels of pro-inflammatory cytokine production, leading to a sub-pathological and presumably also local inflammatory response. A highly inflammatory insult, however, may bypass metalloproteinase-mediated cytokine production, allowing for a stronger and more immediate innate immune response. Such a mechanism would allow the innate immune system to distinguish between basal levels of inflammatory stimuli and more severe challenges which require rapid initiation of inflammation. Further studies will help to elucidate the distinct signaling mechanisms that allow for iRhom2-dependent and -independent regulation of the inflammatory response. Intriguingly, it has been reported that high-dose LPS induces a differential signaling cascade than low-dose LPS. While high-dose LPS activates the nuclear factor kappa-light-chain-enhancer of activated B cells (NF-κB) pathway, low-dose LPS is unable to activate NF-κB but activates the CCAAT/enhancer binding protein delta (C/EBPδ) pathway, leading to a mild and leaky expression of pro-inflammatory mediators (Maitra et al., 2011).

In conclusion, we have shown that low levels of inflammatory stimuli lead to an iRhom2 and metalloproteinase-dependent regulation of cytokine expression, most likely dependent on ADAM17, and that higher concentrations of stimuli lead to a metalloproteinase-independent inflammatory response. These results provide evidence for a novel, previously uncharacterized inflammatory pathway whereby the innate immune response is differentially regulated in an iRhom2/metalloproteinase-dependent manner.

## Data Availability Statement

The original contributions presented in the study are included in the article/supplementary materials. Further inquiries can be directed to the corresponding authors.

## Ethics Statement

The animal study was reviewed and approved by University of Iowa IACUC.

## Author Contributions

JS: conceptualization, methodology, investigation, validation, writing—original draft, review and editing. GD: investigation. CB: conceptualization, writing—review and editing. PI: conceptualization, methodology, investigation, writing—review and editing. TM: supervision, visualization, conceptualization, methodology, writing—review and editing, funding acquisition. All authors contributed to the article and approved the submitted version.

## Funding

This work was funded by the Roy J. and Lucille A. Carver College of Medicine to PI and TM and in part by NIH R01 GM64750 and by DoD Discovery Award W81XWH-13-1-0170 to CB. Additional support was provided by the American Cancer Society (Award Numbers ACS-IRG-18-165-43 and ACS-IRG-15-176-41) and by a Carver Trust Collaborative Pilot Grant through the University of Iowa to TM as well as by a T32 AI007485 to JS.

## Conflict of Interest

CB and the Hospital for Special Surgery have identified iRhom2 inhibitors and have co-founded the start-up company SciRhom in Munich to commercialize these inhibitors. CB and TM hold a patent on a method of identifying agents for combination with inhibitors of iRhoms.

The remaining authors declare that the research was conducted in the absence of any commercial or financial relationships that could be construed as a potential conflict of interest.

## Abbreviations

ADAM17, a disintegration and metalloproteinase 17; BMDM, bone marrow-derived macrophage; CCAAT/enhancer binding protein delta, C/EBPδ; CXCL2, C-X-C motif chemokine ligand 2; DPC333, (((2R)-2-((3R)-3-amino-3(4-[2-methyl-4-quinolinyl) methoxy] phenyl)-2-oxopyrrolidinyl)-N-hydroxy-4-methylpentanamide))); EGF, epidermal growth factor; EGFR, epidermal growth factor receptor; IL, interleukin; iRhom 1, inactive rhomboid 1; iRhom 2, inactive rhomboid 2; LPS, lipopolysaccharide; NF-κB, Nuclear factor kappa-light-chain-enhancer of activated B cells; PBMC, peripheral blood mononuclear cell; TLR, toll-like receptor; TNF, tumor necrosis factor; TNFRSF1A/B, tumor necrosis factor receptors 1 and 2; WT, Wild type.
